# Ventricular Zone Expressed PH Domain Containing 1 (VEPH1): an adaptor protein capable of modulating multiple signaling transduction pathways during normal and pathological development

**DOI:** 10.1186/s12964-019-0433-4

**Published:** 2019-09-09

**Authors:** Theodore J. Brown, Alexandra Kollara, Premalatha Shathasivam, Maurice J. Ringuette

**Affiliations:** 1 Lunenfeld-Tanenbaum Research Institute, Sinai Health System, 60 Murray Street, Box 42, Toronto, ON M5T 3L9 Canada; 2 Department of Obstetrics and Gynecology, University of Toronto, Toronto, ON Canada; 3 Department of Cell and Systems Biology, University of Toronto, Toronto, ON Canada

**Keywords:** VEPH1, Melted, Signal transduction, Adaptor protein, *Drosophila*

## Abstract

Ventricular Zone Expressed PH Domain-Containing 1 (VEPH1) is an 833-amino acid protein encoded by an evolutionarily conserved single-copy gene that emerged with pseudocoelomates. This gene has no paralog in any species identified to date and few studies have investigated the function of its encoded protein. Loss of expression of its ortholog, melted, in *Drosophila* results in a severe neural phenotype and impacts TOR, FoxO, and Hippo signaling. Studies in mammals indicate a role for VEPH1 in modulating TGFβ signaling and AKT activation, while numerous studies indicate VEPH1 expression is altered in several pathological conditions, including cancer. Although often referred to as an uncharacterized protein, available evidence supports VEPH1 as an adaptor protein capable of modulating multiple signal transduction networks. Further studies are required to define these adaptor functions and the role of VEPH1 in development and disease progression.

## Background

Adaptor proteins are critical members of signal transduction cascades, orchestrating the interaction of key members to either augment or reduce signal intensity. Ventricular Zone Expressed PH Domain-Containing 1 (VEPH1) is emerging as an intracellular adaptor protein capable of modulating multiple signal transduction pathways, including TGFβ, mTOR, FOXO, Hippo, and AKT. Much of what is known about the function of this protein is inferred from studies of its ortholog in *Drosophila*; however, expression of VEPH1 is altered in several pathological conditions, including metabolic and neurologic conditions, and cancer. In this review, we summarize what is currently known of the expression and actions of VEPH1 and its orthologs, and propose a model by which VEPH1 may modulate multiple signal transduction pathways.

### Discovery of VEPH1

**Fig. 1:**
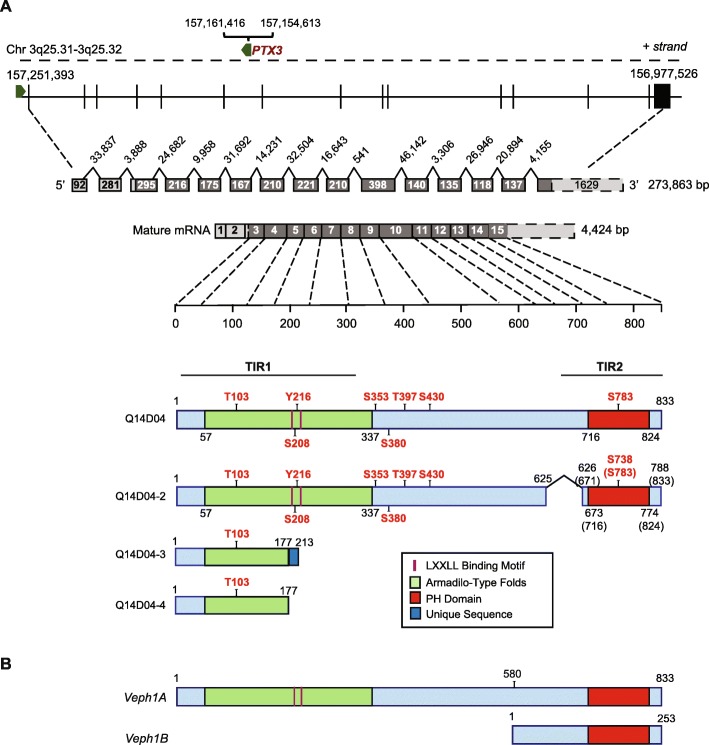
**a** Schematic showing the relationship between the human *VEPH1* gene locus on the minus strand of chromosome 3, primary transcript, mature transcript, and encoded protein based upon data derived from NCBI AceView (http://www.ncbi.nlm.nih.gov/ieb/research/assembly). The protein sequence contains a conserved predicted N-terminal Armadillo-like fold and a C-terminal PH domain (www.ebi.ac.uk/interpro/protein/Q14D04), as well as two LLxxL nuclear receptor binding motifs. Three splice variants of the primary transcript have been reported with their predicted encoded products shown. Potential phosphorylation sites, identified by the Eukaryotic Linear Motif (ELM) resource prediction tool, are indicated with red text. TIR1 and TIR2 indicate sequences corresponding to the human protein that were shown to interact with TGFβ receptor 1 (ALK5). **b** Schematic showing the predicted proteins (Veph1A and Veph1B) encoded by full-length mouse *Veph1* transcript and a reported alternatively spliced variant

Veph1 was designated by Muto et al. in 2004 [1], during a search for candidate genes that contribute to differentiation of neural and glial progenitor cells in the fetal mouse brain ventricular zone. Full-length *Veph1* cDNA encodes an 833-amino acid protein with a C-terminal pleckstrin homology (PH) domain (amino acids 716–824) and a predicted Armadillo-type fold domain (amino acids 57–337) as its only identifiable functional domains (Fig. 1). Four years prior to this publication, Nagase et al. [2] had isolated a similar cDNA sequence, KIAA1692, along with other expressed sequences from size-fractionated cDNA libraries generated from human fetal and adult brains. Based on computer modeling, KIAA1692 was matched to a locus on chromosome 3. Database queries of non-mammalian genomes revealed similarity to an unclassified homolog in *Drosophila*, referred to as CG8624, which localized to *Drosophila* chromosome 3 and was later recognized to be *melted*.

**Fig. 2:**
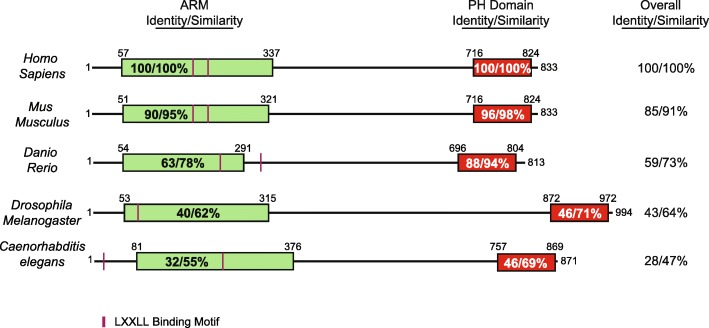
Evolutionary amino acid sequence conservation of VEPH1 from nematodes to mammals. Overall sequence identity and similarity percentages are in reference to the human sequence. Between species, both the N-terminal Armadillo-like domain and the PH domain show the highest amino acid sequence identity and similarity, suggesting an importance to function. No significant sequence similarity or identity was found in animal ancestral to pseudocoelomates

Salzberg et al. [3] had actually identified *Drosophila melted* three years previously, in 1997, in a genetic screen of P-element insertions within chromosome 3 that affected peripheral nervous system (PNS) development. Melted encodes a 994-amino acid protein with both a predicted N-terminal Armadillo-like fold and a C-terminal PH domain, both of which are conserved within phyla (Fig. 2). Embryos homozygous for a *melted* mutation showed an abnormal morphology of PNS neurons, leading the authors to designate the gene ‘melted’ to accentuate the mutant phenotype of the aggregated or fused PNS neuronal cell bodies. Deletion of melted resulted in a 30% reduction in flies reaching maturity, an approximate 10% lower body weight, and a 25 and 40% reduction in fat body and total body triglycerides, respectively [4].

Studies in zebrafish, where Veph1 is expressed in the embryonic brain, including the ventricular zone and otic vesicles, support a role of Veph1 in neural development. Knockdown of Veph1 protein expression in zebrafish embryos using targeting morpholino antisense RNA resulted in impaired midbrain and hindbrain development, as well as a curved spine, impaired ear development, pericardial edema, and disordered lateral strip pigmentation, indicating that Veph1 is required for the normal development of multiple tissues. A recent study found Veph1 amongst several genes with expression in murine neural stem cells at embryonic day (E) 15.5 and 17.5 but not E13.5, suggesting that Veph1 expression may be activated during the attenuation of the proliferative state of neural stem cells [5]. VEPH1 expression was increased during retinoic acid-induced differentiation of human NT2 teratocarcinoma cells into neurons in vitro, further implicating a role for VEPH1 in mammalian neuronal cell differentiation [1]. Despite this, and the pronounced phenotypes in model organisms, targeted disruption of *Veph1* in mice was not associated with an overt phenotype; however, an extensive investigation has not been reported [1].

### Organization of the *VEPH1* gene locus and identifiable domains

Veph1 appears to have emerged with pseudocoelomates and paralogs have not been identified in the genome of any species annotated to date. In humans, the *VEPH1* gene is found on the minus strand of chromosome 3 at q25.31-q25.32, and extends over nearly 274 kilobases. The gene consists of 15 exons and 14 introns, with the coding region extending across exons 3 to 15 (Fig. 1). A similar structural organization exists for the murine *Veph1* locus. Consistent with the complexity of the locus, several transcript variants resulting from alternate RNA splicing have been reported for human *VEPH1* (Fig. 1). It is not known if these variants result in expressed proteins.

The most prominent known feature of VEPH1 is its PH domain. One common function of PH domains is a stimulus-dependent recruitment of molecules to the cell membrane. Consistent with this idea, melted at low concentrations has been shown to bind selectively to phosphatidylinositol-5-phosphate [4], and both the *Drosophila* and human orthologs appear to localize preferentially, but not exclusively, to the plasma membrane [4, 6]. In addition, assessment of the amino acid sequence of VEPH1 with the protein sequence analysis and classification tool (InterPro) of the European Bioinformatics Institute indicates the N-terminal region of VEPH1 contains tandem armadillo-like repeat sequences. These sequences characteristically consist of numerous alpha helices of approximately 40 amino acids arranged in two layers to create a superhelical structure capable of binding large proteins and nucleic acids [7]. Through the combination of these two domains, which are both highly conserved (Fig. 2), VEPH1 would be predicted to function as an adaptor or docking protein capable of binding divergent molecules to alter their cellular localization and/or physical interactions to effect changes in function or cell signaling [8–11].

Use of the eukaryotic linear motif prediction tool (ELM.eu.org) identifies multiple interesting motifs with high conservation score that are characteristic of adaptor proteins. These include 14–3-3 interacting motifs, Forkhead association motifs, and SH2 binding motifs. In addition, two LxxLL nuclear receptor binding motifs separated by 18 amino acids are found in mouse and human sequences with one of these conserved in zebrafish and nematodes (Fig. 2). LxxLL motifs are recognition sequences often found in nuclear receptor co-regulatory proteins that bind activating function-2 (AF-2) regions of steroid hormone nuclear receptors (SHRs) as well as mediate binding to other transcription factors [12]. It remains to be confirmed whether VEPH1 forms functional interactions through these motifs as these may be masked by the tertiary structure of the protein.

### Expression of VEPH1

**Fig. 3:**
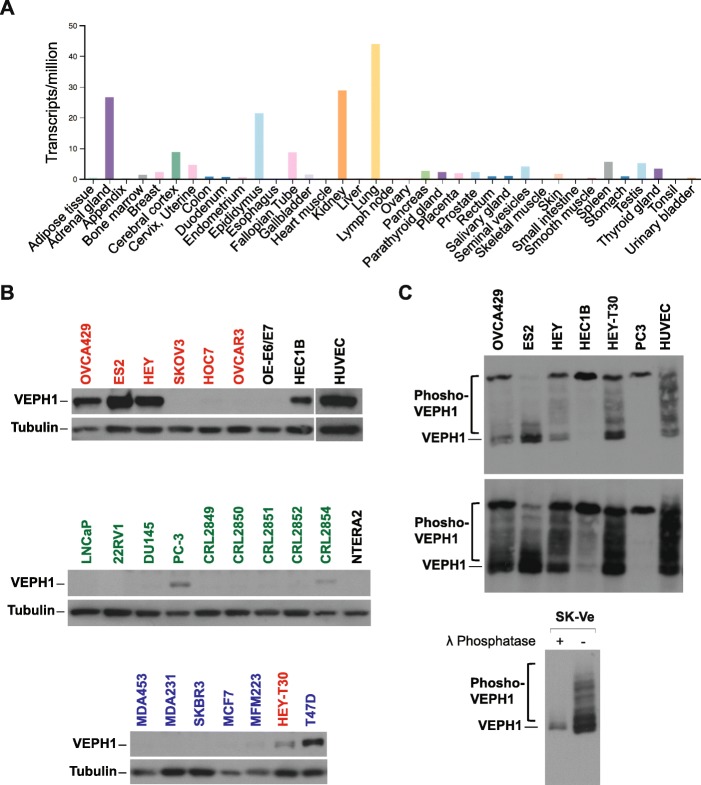
Distribution of *VEPH1* transcripts in human tissue and VEPH1 protein levels in established cell lines. **a** *VEPH1* transcripts in human tissues reported in the human protein atlas (HPA) RNA dataset (proteinatlas.org). **b** VEPH1 protein levels expressed by various human cancer cell lines and non-malignant cell lines indicated by western blotting. Red = ovarian cancer cells, blue = breast cancer cells, green = prostate cancer cells, OE-E6/E7 = immortalized fallopian tube epithelial cells, HUVEC = human umbilical vein endothelial cells, HEC1B = endometrial cancer cells, CRL2854 = immortalized prostate fibroblasts, NTERA2 = testicular cancer cells. Data shown for the six ovarian cancer cell lines replicate our previously reported findings [6]. **c** Differential phosphorylation states of VEPH1 as shown by PhosTag western blot analysis. Shown are two exposure levels (top and middle blots) and results of VEPH1-transfected SKOV3 cells (SK-Ve) treated with or without λ-phosphatase (lower blot)

VEPH1 displays a restricted pattern of expression. As discussed earlier, VEPH1 is expressed in neural tissues during embryogenesis. Data on transcript levels in human tissues reported in the Human Protein Atlas indicate expression in the brain, particularly the cerebral cortex, and strong expression in the pituitary and adrenal gland. High levels of transcripts are also reported for the lung, kidney, reproductive tract structures, and adrenal gland (Fig. 3a). Unfortunately, current commercially available antibodies for VEPH1 are not sufficiently specific to produce accurate indications of protein expression by immunohistochemistry. Among cell lines queried for VEPH1 transcripts, several endothelial, neural, fibroblast, breast, and sarcoma cell lines have been shown to express VEPH1 transcripts (human protein atlas). We have confirmed VEPH1 protein expression in some human ovarian cancer cell lines by western blot analysis [6]. In addition, endogenous VEPH1 protein expression is detectable in HEC1B endometrial cancer cells, PC-3 prostate cancer cells, CRL2854 prostate stromal cells, T47D breast cancer cells, and human umbilical vein endothelial cells (HUVEC) (Fig. 3b). Of the 6 ovarian cancer cell lines examined, three cell lines (HEY, ES2, and OVCA429) known to be aggressive [13] were found to have high levels of VEPH1 expression, whereas VEPH1 expression was not detected in the remaining three less aggressive cell lines (SKOV3, OVCAR3, and HOC7).

**Fig. 4:**
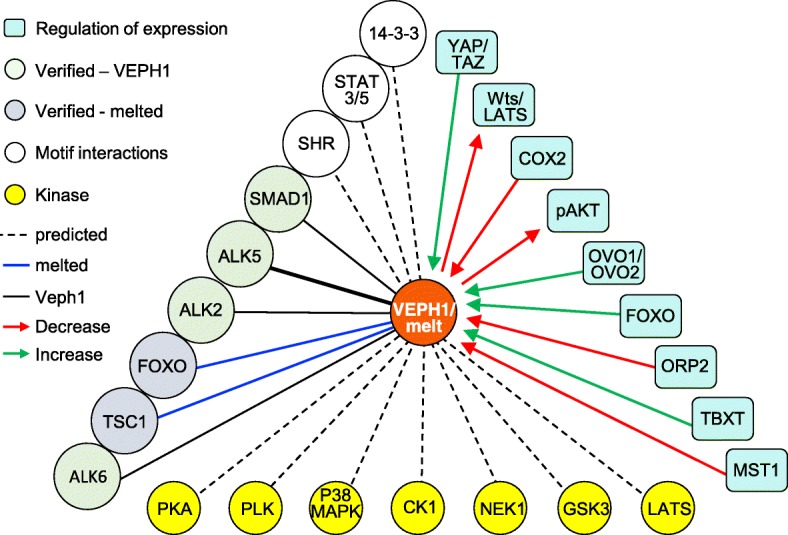
Summary of known and predicted interactions of VEPH1

Phosphosite prediction modeling shows 14 potential phosphorylation sites within VEPH1, some of which are depicted in Fig. 1a. Consistent with this, we have found divergent patterns of the phosphorylated state of VEPH1 in several human cell lines expressing the protein endogenously, as determined by phosphate-affinity polyacrylamide electrophoresis (Fig. 3c). For example, most of the VEPH1 expressed by ES2 ovarian cancer cells is in an unphosphorylated state, whereas that expressed by PC-3 prostate cancer cells and HEC1B endometrial cancer cells is hyperphosphorylated. In comparison, VEPH1 expressed in HUVEC cells, which in vitro recapitulate many aspects of vascular biology, exhibits the greatest heterogeneity in its phosphorylated state. Examples of interacting kinases identified by the eukaryotic linear motif prediction tool with high conservation score include GSK3, casein kinase 1 (CK1), LATS, Nim-A related kinase 2 (NEK1), polo-like kinases (PLK), protein kinase A (PKA), and p38 MAPK (Fig. 4). The different patterns of phosphorylation states likely reflect differential activity of expressed kinases/phosphatases in these cells and could impact protein interactions and the activity of VEPH1 in a cell and situational context.

### *Drosophila* melted as a modifier of cell signal transduction pathways

Melted has been shown to modulate FoxO, target of rapamycin (TOR), and Hippo signaling during *Drosophila* development [4, 14, 15]. The FoxO family of transcription factors regulate several genes involved in growth suppression, DNA repair, amelioration of oxidative stress, and apoptosis. Growth factor signaling leads to phosphorylation of FoxO transcription factors by AKT, leading to FoxO binding with 14-3-3 proteins and cytoplasmic localization, thereby promoting cell cycle progression and survival. Cell proliferation and survival are also regulated by the TOR pathway, which is similarly controlled by activated AKT. TOR is a ser/thr kinase that forms a complex with RAPTOR and mLST8 (TOR complex 1; TORC1) which acts to increase protein synthesis and cell proliferation. TORC1 is inhibited by the GTPase activating protein tuberous sclerosis complex 2 (TSC2). In its non-phosphorylated state, TSC2 binds to and is stabilized by TSC1. Activated AKT phosphorylates TSC2, leading to its dissociation from TSC1 and degradation, resulting in activation of TORC1. TOR also forms a complex with RICTOR, mSIN1, and mLST8 (TORC2). In contrast to TORC1, TORC2 is stimulated by TSC1/TSC2 and activates AKT [16]. In fact, maximal activation of AKT requires the combined activity of TORC2 and PDK1; thus, through its regulation of TSC2, AKT acts to self-limit its activation status. Melted has been reported to interact with FoxO and TSC1, resulting in their sequestration at the cell membrane and attenuation of FoxO signaling and promotion of TORC1 activity [4]. Although not examined, one would predict that decreased TSC2/TSC1 activity would also lead to diminished TORC2 and decreased phosphorylation of AKT. Consistent with this prediction, expression of VEPH1 in SKOV3 ovarian cancer cells is associated with decreased levels of AKT [17].

Jukam and Desplan [14] studied the role of melted in the context of neural progenitor differentiation to specific sensory neuronal phenotypes using the *Drosophila* eye as a model system. The *Drosophila* eye consists of approximately 800 omatidia, each containing eight photoreceptor neurons (R1-R8). The differentiation fate of R8 neurons, which are similar to vertebrate cone cells, is regulated by opposing expression of melted and warts, the ser/thr kinase downstream effector of Hippo/Mst. Melted and warts each favor divergent differentiation pathways. Key to the ultimate differentiation pathway selected is the mutual inhibitory regulation of these two proteins, where melted acts to repress the expression of warts, resulting in increased nuclear retention of yorkie, whereas warts acts to inhibit yorkie to diminish expression of melted [14, 18]. A unique aspect of this mechanism is the regulation of warts activity through its expression levels rather than through its activation by phosphorylation. The human orthologs of yorkie, YAP1 and TAZ, are transcriptional coactivators of the TEA domain (TEAD) family of transcription factors. Consistent with the finding that yorkie regulates melted expression, TEAD4 has been demonstrated to bind to the human *VEPH1* gene by ChIP analysis [19].

Melted was also initially described as being involved in insulin/PI3K signaling in *Drosophila* where it was found to promote tissue growth and fat storage within the larval fat body, effects that were shown to require the presence of the PH domain [4]. Subsequently, melted was identified as a core component of the insulin signaling pathway in *Drosophila* S2R+ macrophage-like hemocyte-derived cells [20]. Melted transcript levels were rapidly and transiently increased by insulin treatment, and melted protein was found to interact with multiple AKT and ERK regulators [20].

Studies exploring a role for VEPH1 in insulin signaling have not been reported; however, based upon sequence similarity and the fact that human VEPH1 could rescue the reduced growth effects observed in *melted*-null flies, it is possible that VEPH1 functions similarly in mammalian insulin signaling. In a meta-analysis study comparing gene expression patterns in peripheral lymphomononuclear cells isolated from patients with gestational, type-1, or type-2 diabetes, Collares et al. [21] identified VEPH1 as 1 of 7 genes highly up-regulated across all three diabetes mellitus subtypes. Expression was 2-fold higher in gestational diabetes than in type-1 diabetes, which in turn was 2-fold higher than in type-2 diabetes. The functional significance of the differential expression of VEPH1 in diabetes, particularly gestational diabetes, warrants further investigation.

### VEPH1 modulation of TGFβ signaling

Consistent with the impact of *Drosophila* melted on signaling pathways, expression of *VEPH1* cDNA in ovarian cancer SKOV3 cells was shown to affect FoxO, mTOR, and YAP/TAZ signaling networks by pathway analysis of differentially expressed genes identified by microarray-based gene expression profiling [6]. In addition, an impact on TGFβ signaling was identified.

The PH domain of murine VEPH1 (FLJ12604) had been used previously in a screen to identify key domains and proteins that interact with type-I and -II receptors of the TGFβ ligand superfamily [22]. This superfamily of cytokines and growth factors, which include bone morphogenic proteins (BMPs), activins, inhibins, and nodal, in addition to TGFβ isoforms, regulates nearly all aspects of development, with dysregulation of these signaling pathways involved in multiple pathologies. Signaling by these ligands is initiated by binding of the ligand to type-II and type-I ser/thr kinase receptors at the plasma membrane, which brings the two receptor types in close proximity to one another. As a result, the type-I receptor is phosphorylated and activated by the constitutively active type-II receptor. Canonical signaling is mediated by type-I receptor phosphorylation and activation of receptor SMADs (SMADs 1, 2, 3, 5, or 8) which then dimerize with SMAD4 and translocate to the cell nucleus to function at response elements within the regulatory elements of target genes. The genes regulated are dependent upon the specific receptor SMADs activated, their level of nuclear accumulation and retention, and the presence of co-regulatory proteins and other transcriptional factors. The PH domain of murine VEPH1 was found to interact with type-I receptors ALK2, ALK5, and ALK6 as well as with SMAD1 [22].

ALK5 is the type-I receptor activated by TGFβ and signals canonically by activating SMAD2 and SMAD3. We have confirmed full-length VEPH1 interacts with ALK5, resulting in retention of SMAD2 at the receptor and decreased nuclear accumulation and retention of both SMAD2 and SMAD3, which in turn results in decreased TGFβ target gene expression [6]. While VEPH1 expression did not affect SMAD2 phosphorylation levels, it resulted in decreased TGFβ-induced phosphorylation of SMAD3 and total SMAD3 levels. Notably, both the armadillo repeat domain containing N-terminal region and the PH domain of VEPH1 interact with ALK5. Expression of the N-terminus TGFβ receptor I interacting region (TIR1) inhibited TGFβ-induced SMAD2/3 signaling similar to that of full-length VEPH1. In contrast, the isolated PH domain (TIR2) markedly enhanced TGFβ-induced signaling [6]. Interestingly, a splice variant of Veph1 (isoform-B) expressed in mouse (but not reported for humans) is predicted to encode a truncated protein consisting of the C-terminal domain of Veph1 analogous to the TIR2 sequence (Fig. 1b). Using PCR, we found that this alternately spliced transcript is expressed in mice but only during fetal development [23]. The precise embryonic tissue distribution and whether this transcript is translated into functional protein remains to be determined. However, based upon the differential activity of TIR2 alone versus that of TIR2 integrated into full-length VEPH1, we predict that mouse Veph1 isoform B would act to amplify TGFβ signaling during embryonic development and may function as a dominant negative of Veph1 isoform A. These findings also have implications for the development of therapeutic modulators for use in pathological states where altered TGFβ signaling is an underlying driver.

TGFβ is a ubiquitous cytokine impacting multiple developmental and physiological processes with pathological effects resulting from its dysregulation [24]. For example, excessive TGFβ signaling is a well-known trigger of tissue fibrosis. TGFβ activates tissue fibroblast differentiation to myofibroblasts, which secrete large amounts of collagens and fibronectins, resulting in a dense fibrotic matrix. VEPH1 is decreased in idiopathic human pulmonary fibrosis and is similarly decreased in bleomycin-induced pulmonary fibrosis in a rat model [25]. Thus, decreased expression of VEPH1 in fibrotic tissue is consistent with its attenuating impact on canonical TGFβ signaling activity. On the other hand, insufficient TGFβ signaling can result in heightened immune function and autoimmunity, and may contribute to neurodegenerative diseases. Multiple studies suggest Parkinson’s and Alzheimer’s diseases are triggered or exacerbated by pro-inflammatory signaling within the central nervous system and are associated with insufficient TGFβ signaling [26–28]. Development of therapeutics based upon divergent VEPH1 TIR1 and TIR2 activities might allow dampening or enhancement of endogenous SMAD-dependent TGFβ signaling to restore appropriate signaling levels.

Since other identified candidate interacting partners of Veph1 are involved with BMP signaling, further studies should focus on the impact of VEPH1 on signaling by other TGFβ superfamily members. SMAD signaling pathways have been implicated in stem cell regulation and control cell fate decisions. BMPs promote an undifferentiated state by upregulating inhibitor of differentiation (Id) proteins through SMAD1 activation, whereas Actvin/Nodal/TGFβ-regulated SMAD2/3 promotes stem cell self-renewal. As a potential broad-spectrum modulator of the TGFβ superfamily signaling network, VEPH1 could influence progenitor cell amplification and fate decisions.

TGFβ regulates trophoblast invasion during placentation with dysregulated expression and signaling contributing to pre-eclampsia, a common and potentially lethal disorder in pregnancy [29–31]. Although the precise mechanisms involved in development of pre-eclampsia are incompletely understood, the disease is largely considered to be the result of inadequate invasion of trophoblast cells into the uterine placental arteries during placentation and by impaired VEGF signaling and vasoconstriction, resulting in poor placental perfusion and increased maternal blood pressure. A recent study comparing RNA transcripts in early pregnancy peripheral blood cells from 16 women who later developed pre-eclampsia and 16 women who went on to normotensive pregnancies identified 86 up-regulated genes and 161 down-regulated genes in women destined to develop pre-eclampsia [32]. VEPH1 was among the top 7 up-regulated genes in the pre-eclampsia cohort. Given the impact of VEPH1 on TGFβ signaling and on VEGFA expression, [6, 17], VEPH1 may contribute to impaired placental development by repressing pro-angiogenic signaling.

### VEPH1 identified as cargo protein of exosomes derived from diverse cell types

An exciting emerging mechanism for cell-cell communication is the release and uptake of exosomes, which are small (30–100 nm diameter) lipid bilayer extracellular vesicles containing cargo that impact signaling pathways and activities of recipient cells. Proteomic investigations have identified VEPH1 as a component of exosomes originating from various cell types including trophoblast cells, cancer associated fibroblasts, and mesenchymal stem cells [33–35]. Exosomes derived from two in vitro models of trophectoderm cells, JEG3 human choriocarcinoma cells and HTR8/SVneo extravillous trophectoderm cells, are taken up by vascular smooth muscle cells associated with uterine spiral arteries to enhance their migration during placentation [35]. Placental mesenchymal stem cells similarly produce exosomes that promote placental microvascular endothelial cell migration and tube formation, with exosome production augmented under hypoxic conditions. VEPH1 was identified in exosomes produced by these cells under both hypoxic and normoxic conditions [34]. These findings raise the question of a role for VEPH1 in modulating uterine and placental endothelial cell signaling and vascular remodeling necessary for efficient placental development and function.

Exosomes have also emerged as important promoters of tumor cell migration and invasion. For example, exosomes released from Muloney Sarcoma Virus-transformed mouse embryonic fibroblast cells (L cells) promote breast cancer cell motility and invasion by providing molecular components of the autocrine WNT11 planer polarity signaling pathway [33]. VEPH1 was identified as one of the proteins present within these exosomes. Despite being present in exosomes from diverse sources, it remains to be determined as to whether VEPH1 contributes to exosome production or to changes in signaling events in recipient cells.

### Altered VEPH1 expression in multiple cancer types

Evidence of differential expression of VEPH1 has been reported in diverse cancer types (Table 1), although this evidence is often only presented in supplemental files with few studies addressing the function of VEPH1 in these cancers. In addition, mutations and loss of heterozygosity in VEPH1 have been associated with invasive breast cancer and genome-wide association studies have produced a growing list of single nucleotide polymorphisms within the *VEPH1* locus in a variety of malignant tumors [38, 39, 43, 52] (https://hive.biochemistry.gwu.edu/biomuta/proteinview/Q14D04). The impact of these variations on function remains unexplored.

**Table 1:** Studies reporting altered expression or variants of VEPH1 in cancer

Cancer	Predicted positive or negative impact	Reference	Notes
Adrenocortical cancer	Positive	[36, 37]	VEPH1 expression is one of 4 genes predictive of increased overall survival
Breast cancer	Unknown impact of mutation	[38]	Identified VEPH1 as a cancer gene based on mutation rate
Breast cancer	Negative	[39]	LOH of the VEPH1 locus
Chordoma	Negative	[40]	Target gene of Brachyury (TBXT)
Colorectal cancer	Negative/Positive	[41]	VEPH1 expression was increased 7-fold in DLD-1 colorectal cancer cells expressing FOXO3a
Gastric cancer	Positive	[42]	Decreased > 50% in early gastric cancer relative to normal tissue
Hepatocellular cancer	Unknown impact of SNPs	[43]	Region within intron 4 is one of nine susceptibility loci associated with hepatocellular cancer
Hepatocellular cancer	Positive	[44]	VEPH1 expression increased by ORP2 knockdown
Lung squamous cell cancer	Positive	[45]	VEPH1 transcripts decreased nearly 80% relative to non-tumor lung tissue
Multiple myeloma bone marrow MSCs	Negative	[46]	May be involved in establishing a microenvironmental niche favoring cancer progression
Osteosarcoma	Positive	[47]	Decreased expression in COX2-overexpressing osteocarcinoma cells
Ovarian cancer	Positive	[17]	VEPH1 expression resulted in decreased xenograft tumor progression
Ovarian cancer	Negative	[48, 49]	Amplification of the *VEPH1* locus and expression
Prostate cancer	Negative	[50]	Decreased by STK4/MST1 overexpression in cell lines
Prostate cancer	Unknown impact of splice variant	[51]	Increased alternate splicing of VEPH1 associated with EMT

In a study published in 2014, Ragazzon et al. [36] examined the genetic heterogeneity associated with highly variable outcome among patients diagnosed with adrenocortical carcinoma (ACC). Although rare, this cancer is highly aggressive with a 5-year survival rate below 35%. However, there is a pronounced variability in prognosis and outcome that is poorly understood, which may relate to the intrinsic biology of the tumors and/or to different mechanisms of tumor development [37]. There are two genetic syndromes associated with ACC: Beckwith-Wiedemann syndrome, due to IGF2 overexpression, and Li-Fraumeni Syndrome, due to inactivating p53 mutations. In addition, there are sporadic cases that appear to be monoclonal. IGF2 overexpression is common, occurring in ~ 85% of cases, whereas p53 mutations are present in ~ 35% of sporadic cases. Molecular profiling shows very different gene expression between benign and malignant adrenal cortical tumors. Two cluster groups of ACC were identified based on their gene expression profiles and overall survival. Patients with tumors within cluster 1 had a 5-year survival rate of 20% whereas those with tumors in cluster 2 had a 5-year survival rate of more than 90%. Further study of these two cluster cohorts among the 51 ACC cases identified 41 gene candidates for predicting outcome. Of these genes, VEPH1 along with MCM5, PINK1, and SLC2A1 genes provided the best discriminatory power, with VEPH1 expression decreased by 90% in the aggressive cancers (cluster 1) compared to those with better survival.

Consistent with these studies suggesting increased VEPH1 expression associates with better outcome, we found that expression of ectopic VEPH1 in human ovarian cancer SKOV3 cells resulted in decreased tumor progression [17]. Somewhat surprisingly, VEPH1 expression did not appear to alter tumor cell proliferation but rather increased necrotic regions within the tumor. Further in vitro studies indicated that VEPH1 expression in these cells resulted in decreased expression of pro-angiogenic factors *VEGFA* and *IL-8*. This decrease could be at least partially explained by decreased AKT activation; however, the underlying mechanisms remain to be defined.

Some studies offer support for the idea that VEPH1 may be involved in cancer initiating events. Amplification of the *VEPH1* gene locus associated with increased transcript levels has been reported in ovarian cancers [48, 49], and query of the TCGA database indicates potential amplification in multiple other cancers [6]. However, in gastric cancer, *VEPH1* expression is decreased by > 50% in early stage disease [42], suggesting loss of VEPH1 expression may enable malignant transformation or survival of these cells. *VEPH1* transcripts were also found to be decreased in lung squamous cell carcinoma relative to non-tumour lung tissue in microarray studies performed on either macro-dissected and laser-capture micro-dissected tissues [45].

Levels of *VEPH1* have been shown to be affected by expression of various drivers of cancer progression. Increased *VEPH1* expression is driven by Brachyury/TBXT, a T-box transcription factor highly expressed in chordoma, a rare cancer derived from notochord remnants [40]. Given the central role of Brachyury in chordoma, this finding raises the possibility that VEPH1 may promote this cancer. In contrast, *VEPH1* expression is decreased by STK4/MST1 overexpression in prostate cancer cell lines [50]. STK4 is the human ortholog of Hippo that activates LATS to result in decreased nuclear YAP and TAZ accumulation; thus, functioning to suppress tumor progression. This finding is consistent with the reported effect of Yorkie on melted expression in *Drosophila* [14] and the finding by Lim et al. [19] of binding of TEAD4, the transcription factor partner of the YAP and TAZ co-activators, to the *VEPH1* gene locus in gastric cancer cells.

*VEPH1* was found to be among the genes with the most strongly up-regulated expression in Huh7 hepatocellular carcinoma cells upon disruption of *OSBPL2/ORP2* (Oxysterol Binding Protein-Like 2) by CRISPR-Cas9. *OSBPL2* encodes a lipid-sensing protein that regulates bidirectional cholesterol trafficking between the endoplasmic reticulum, lipid droplets, and plasma membrane [53], but also plays an essential role in regulating actin cytoskeletal dynamics [44]. Disruption of OSBPL2 in Huh7 cells resulted in abnormal F-actin formation, leading to impaired lamellipodia formation and cell migration [44]. The mechanism by which *OSBPL2* expression impacts *VEPH1* expression and whether decreased VEPH1 is involved in mediating OSBPL2 effects remain an untested possibility.

A study with osteosarcoma cells also shows decreased *VEPH1* expression associated with a known driver of disease progression. Proliferation and invasion of osteosarcoma cells is stimulated by cyclooxygenase 2 (COX2) and subsequent prostaglandin production. Overexpression of COX2 in U2OS osteosarcoma cells resulted in down-regulation of 20 genes, including *VEPH1*, suggesting that VEPH1 may be a negative regulator of osteosarcoma cell proliferation or invasion by up-regulated COX2 expression [47]. Interestingly, a study by Roca et al. [51] found an isoform of *VEPH1* was up-regulated more than 4-fold by Ovo-Like Transcriptional Repressor 1 and 2 (OVO1/OVO2) in prostate cancer cells. Overexpression of OVO1/OVO2 in mesenchymal-like prostate cancer cells promoted transition to an epithelial-like phenotype to allow colony formation at metastatic sites. Altogether these studies raise the possibility that VEPH1 may augment signaling networks involved in prostate cancer progression and may be involved in mesenchymal-epithelial cell transitions that can impede metastasis while promoting expansion of lesions formed by metastatic cells.

Tenbaum et al. [41] found *VEPH1* expression to be increased more than 7-fold by overexpression of FOXO3a in DLD-1 colorectal cancer cells. FOXO3a is generally considered to act to suppress tumor progression by inducing cell cycle arrest and apoptosis. However, FOXO3a binds nuclear β-catenin and the joint action of nuclear β-catenin and FOXO3a strongly promotes metastasis, with β-catenin providing protection against FOXO-induced apoptosis [41]. As AKT mediated phosphorylation of FOXO results in its cytoplasmic localization, our studies showing decreased levels of AKT activation by VEPH1 [17] would predict increased nuclear FOXO localization, resulting in a feed-forward increase in VEPH1 expression and FOXO target gene expression. While FOXO3a generally acts to cause decreased growth and survival of cancer cells, increased nuclear FOXO in the presence of increased WNT signaling activity acts instead to further promote cancer cell metastasis. Thus, the potential impact of VEPH1 on cancer progression may be altered in the context of nuclear β-catenin levels.

Changes to the microenvironment within tissue stem cell niches can have determining effects on renewal vs. progenitor fate decisions and can lead to both cancer initiation and progression. Bone marrow mesenchymal stem cells form an integral part of the bone marrow microenvironment and modulates the survival and growth of malignant plasma cells (multiple myeloma) [54]. Gene expression profiles of these stem cells indicate they are molecularly distinct in patients diagnosed with multiple myeloma as compared to healthy donors [46]. *VEPH1* was among the 145 differentially expressed genes identified, with a 3-fold higher expression in stem cells from multiple myeloma patients. It remains to be determined whether this change in *VEPH1* expression has direct consequences on the activities of the bone marrow mesenchymal stem cells or whether this alters their modulation of associating malignant plasma cells.

### Concluding remarks

**Fig. 5:**
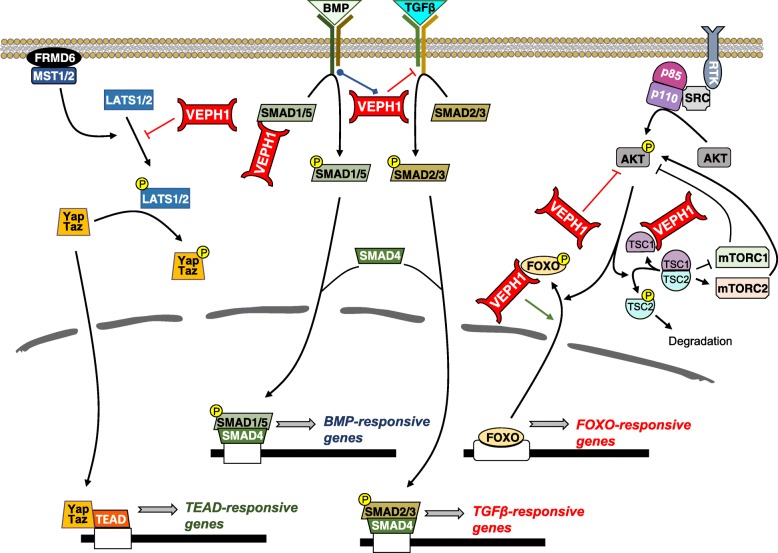
Schematic model showing VEPH1 adaptor function impacting TGFβ, BMP, HIPPO, and AKT signaling networks based upon interactions shown for *Drosophila* melted and mammalian VEPH1, as depicted in Fig. 4

VEPH1 is encoded by an evolutionary conserved single-copy gene that emerged with pseudocoelomates with no known paralog in any species. This complex gene locus is amplified in several cancers and is susceptible to alternate splicing of primary transcripts. Studies in non-mammalian model organisms show a pronounced developmental phenotype resulting from decreased or lack of expression; however, *VEPH1* disruption in mice did not result in an overt phenotype. VEPH1 transcript expression is highly restricted in adult murine tissues, with an absence of information on expected resultant protein expression in these tissues. Based upon what is known of its *Drosophila* ortholog, established and predicted interactions, regulation, and effects on cell signal transduction pathways (summarized in Fig. 4), VEPH1 is emerging as an adaptor protein capable of modulating several cell signaling networks (summarized in Fig. 5). A growing list of studies show altered expression levels of VEPH1 associated with various disease states, with studies in cancer indicating potential pro- and anti-tumorigenic activities.

Studies are required to fully determine the precise mechanistic activities of VEPH1 as a novel adaptor protein:

1. Impact of loss of VEPH1 in mammalian models: Human VEPH1 has been shown to inhibit SMAD-dependent canonical TGFβ signaling by interacting with ALK5 through its N-terminus armadillo-like repeat region. In contrast, the PH domain of VEPH1 amplified TGFβ-induced signaling through an undefined mechanism. A splice variant of VEPH1, encoding for the PH domain (Veph1-B) is expressed during murine organogenesis. This splice variant would likely have a different translational start site and 5′ untranslated region requiring 5′-RACE (rapid amplification of cDNA ends) to verify. The currently available murine model for Veph1 disruption would not have likely impacted expression of this variant. The characterization of tissue-specific Veph1 protein isoform expression throughout murine development, combined with targeted disruption of Veph1-B isoform expression may reveal an overt phenotype. Moreover, although disrupting full-length VEPH1 (VEPH1-A) expression did not produce an identified phenotype, a carefully executed study incorporating aging and application of various stressors may reveal physiologic consequences to loss of expression.
2. Identification of interacting partners of VEPH1 responsible for neural dysplasia in model organisms: In contrast to the mouse model, disruption of VEPH1 in non-mammalian model organisms compromised neural development. As discussed, a strong neural phenotype was shown in zebrafish treated with VEPH1-targeting morpholinos. Using this model organism, an exploration of the contribution of the PH domain and armadillo-repeat regions of VEPH1 to this phenotype using CRISPR-Cas9-generated deletion/mutagenesis approaches and rescue using mRNA injection of human sequences should be conducted. This would be a first step toward identifying candidates from protein and/or lipid interactor screens of VEPH1 that might meditate the neural phenotype in lower organisms but be compensated for in mammals.
3. Impact of VEPH1 phosphorylation: The function of VEPH1 as an adaptor protein is likely influenced by its phosphorylation state. Multiple potential phosphorylation sites within VEPH1 have been predicted with experimental evidence to support some of these sites. Further verification of phosphorylation sites can be performed using liquid chromatography-tandem mass spectroscopy, followed by assessing the impact of substitution for key serine/threonine/tyrosine amino acids on signaling pathways.
4. Tumorigenesis and vascularization: The impact of VEPH1 on multiple cell signaling pathways implicated in tumor initiation and progression, together with reports of altered VEPH1 expression in multiple cancers indicates a potential role in tumor progression. In an initial test of this, we found that VEPH1 expression in an ovarian cancer cell line did not impact the ability of these cells to form tumors but slowed tumor progression relative to those formed by mock-transfected cells. This slowed progression was attributed to increased areas of necrosis and decreased blood vessel content, consistent with decreased VEGF levels^17^. Further work is needed to define the impact of VEPH1 expression on tumor progression, including tumor expansion, angiogenesis, and metastasis using orthotopic syngeneic models.

The impact of VEPH1 in development and disease progression appears to be multifaceted, with effects dependent upon developmental or disease stage as well as on the combination of active signaling networks. In addition to providing needed information on VEPH1 as an adaptor protein, these studies may reveal therapeutic potential of targeting or modifying its activity in various disease states.

## Data Availability

Data sharing is not applicable to this article as no datasets were generated or analysed.

## Abbreviations

ACC: Adrenocortical carcinoma
AF-2: Activating function 2
AKT: Ak strain transforming
ALK: Activin-like receptor kinase
BMP: Bone morphogenic protein
Cas9: CRISPR-associated protein 9
ChIP: Chromatin immunoprecipitation
CK1: Casein kinase 1
COX2: Cyclooxygenase 2
PCR: Polymerase chain reaction
CRISPR: Clustered regularly interspaced short palindromic repeats
C-terminal: Carboxy terminal
DNA: Deoxyribronucleic acid
E: Embryonic day
FOXO: Forkhead box class O
GSK3: Glycogen synthase kinase 3
HUVEC: Human umbilical vein endothelial cells
Id: Inhibitor of differentiation
IGF2: Insulin-like growth factor 2
IL-8: Interleukin 8
LATS: Large tumor suppressor kinase
MCM5: Minichromosome Maintenance Complex Component 5
mLST8: MTOR Associated Protein, LST8 Homolog
mSIN1: Stress-Activated Map Kinase Interacting Protein 1
MST1/2: Mammalian STE20-like 1/2
mTOR: Mechanistic target of rapamycin
NEK1: Nim-A related kinase 2
nm: Nanometre
N-terminal: Amino terminal
OSBPL2: Oxysterol binding protein-like 2
OVO1/2: Ovo-Like Zinc Finger 1/2
PH: Pleckstrin homology domain
PINK1: PTEN induced kinase 1
PKA: Protein kinase A
PLK: Polo-like kinase
PNS: Peripheral nervous system
RACE: Rapid amplification of cDNA ends
RAPTOR: Regulatory associated protein of TOR
RNA: Ribonucleic acid
ser: Serine
SH2: Src homology 2
SHR: Steroid hormone receptors
SLC2A1: Solute Carrier Family 2 Member 1 (Glut1)
SMAD: Mothers Against Decapentaplegic Homolog
STK4: Serine/Threonine Kinase 4
TAZ: Tafazzin
TCGA: The cancer genome atlas
TEAD: TEA domain family member
TGFβ: Transforming growth factor beta
thr: Threonine
TIR1: TGFβ receptor-interacting region 1
TIR2: TGFβ receptor-interacting region 2
TOR: Target of rapamycin
TORC1: TOR complex 1
TORC2: TOR complex 2
TSC1: Tuberous sclerosis 1
TSC2: Tuberous sclerosis 2
VEGFA: Vascular endothelial growth factor isoform A
VEPH1: Ventricular zone expressed PH domain-containing protein 1
WNT: Wingless-related integration
YAP: YES associating protein
